# Editorial: LINAC-based stereotactic radiosurgery in daily practice

**DOI:** 10.3389/fonc.2023.1306329

**Published:** 2023-11-29

**Authors:** Árpád Kovács, Ferenc Lakosi

**Affiliations:** ^1^ Department of Oncoradiology, Faculty of Medicine, University of Debrecen, Debrecen, Hungary; ^2^ Dr. József Baka Diagnostic, Radiation Oncology, Research and Teaching Center, “Moritz Kaposi” Teaching Hospital, Kaposvár, Hungary

**Keywords:** LINAC, SBRT, daily, SRT, practice

How to ensure precision-targeting of the tumor and the attainment of biological effectiveness while sparing normal tissues remains one of the most crucial technical and clinical questions in the domain of local cancer treatment modalities using ionizing radiation, within the continuously changing and improving oncological treatment landscape. In recent decades, besides dedicated stereotactic radiosurgery (SRS) methods and procedures (gamma knife-based intracranial SRS, cyber knife-based intra- and extracranial SRS, stereotactic radiotherapy (SRT), and stereotactic body radiotherpay SBRT), LINAC-based stereotactic radiosurgery and radiotherapy has emerged as a groundbreaking technology that is reshaping daily oncology practice. With its ability to deliver highly precise radiation doses to well-defined tumor regions within and outside the brain while sparing surrounding healthy tissues, and with the wide range of accessibility and availability of LINAC-based SRT, SBRT has garnered significant attention in local treatments in modern oncology.

This editorial delves into the feasibility and clinical impact of imaging and treatment delivery methods, technological advancements in these domains, and the future of LINAC-based stereotactic irradiation methods in modern complex oncology care. Significant technical development has been achieved in the last decade, providing access to a wide and variable range of LINAC-based radiosurgery and radiotherapy methods. Several prospective multicentric clinical trials have proven the advantage of stereotactic radiotherapy (stereotactic ablative radiotherapy, stereotactic body radiotherapy, fractionated stereotactic radiosurgery, and radiotherapy) of single and oligometastatic cancers in different locations, both intra- and extracranially. Recent research on novel imaging methods, such as new PET tracers (PSMA, MET, FDOPA, FMISO), 4D CT, PET-CT, PET-MR, and advanced dedicated sequences of MRI, has provided superior accuracy in tumor definition and intra-fractional target motion management. Fractionated stereotactic radiotherapy and radiosurgery delivery enable the use of daily high-precision adaptive radiation approaches. Optimization efforts for the standard operating procedures in stereotactic radiotherapy will result in improvement of its effectivity and the therapeutic index. To gain deeper insight into LINAC-based stereotactic radiosurgery procedures, we highlight recent articles published in the Research Topic *“LINAC-Based Stereotactic Radiosurgery in Daily Practice*” in *Frontiers in Oncology*. These studies provide valuable data and insights into the technology’s clinical applications and outcomes. We present the imaging, clinical, technological, and innovative findings related to this Research Topic and acknowledge the researchers contributing to this work.

## 1 Imaging

This Research Topic highlights imaging procedures focusing on MR-based target definition of SRS procedures (Huang et al.) and modern prostate-specific membrane antigen-PSMA PET-based imaging of tumor progression (Varga et al.). Conventional MR imaging is the basic method in all intracranial SRS procedures. In the paper by Huang et al., the authors noted the high impact of improved MR resolution on target definition and also on reduced inter-observer variability. Varga et al. reported their results on the effect of PSMA PET information in improving prostate tumor staging and detection of low-volume prostate tumor progression.

## 2 Radiobiology, feasibility, and dosimetry

Feasibility and various dosimetric concepts must be studied and clarified in the design of LINAC-based stereotactic treatments. Tang et al. presented a study focusing on single- and multiple-isocenter LINAC-based stereotactic body radiation therapy for multiple liver metastases. They focused on the VMAT-based method of SBRT planning and treatment delivery. They concluded that there are advantages in terms of total treatment time reduction, improved patient comfort, and improved plan quality when using advanced planning and single-isocenter VMAT-based treatment delivery methods. In their own study, Wu et al. investigated the use of volumetric-modulated arc therapy (VMAT) with flattening-filter-free (FFF) beams (the FFF-based VMAT technique) in SBRT. They concluded that the FFF beam technique for lung SBRT with VMAT results in a better dose fall-off, better dose-sparing of OAR, lower NTCP of the lung, and a shorter beam on-time compared with the FF beam technique.

## 3 Clinical results

LINAC-based SRS and SRT are used in various intra- and extracranial clinical indications. In this Research Topic, a report by Caivano et al. of a retrospective mono-institutional study in a large cohort of patients with lymph node metastases treated by LINAC-based SBRT concluded that LINAC-based SBRT is a good therapeutic option in light of survival, patient safety, and tolerance. Beyond these promising results, the authors recommended multicentric prospective studies to clarify the importance of tumor volume, tumor burden, lesion size and site, and applied dose as predictive factors in SBRT.

Another single-arm phase II study by Nguyen et al. reported the promising results of stereotactic focal radiotherapy in treating low-risk prostate cancer patients. In this specific stereotactic indication, the focus of the procedures (radical therapy versus active surveillance) is more on preserving patients’ quality of life (QOL), rather than on improving survival. The authors concluded that a new, innovative robotized approach to focal SBRT is a feasible, well-tolerated method that preserves the patient’s QOL and can challenge active surveillance.

## 4 Future perspectives

Beyond conventional CT- or CBCT-controlled LINAC systems, MR-LINAC-based stereotactic methods are approaching. A novel LINAC unit using MRI as an image guidance method is opening up new dimensions in clinical practice. Tallet et al. presented their results on the added value of using MRI-LINAC in treatment of liver tumors by SBRT. The authors concluded that using MRI as an IGRT tool for SBRT allows for the significant reduction in the volume of normal healthy liver parenchyma to be irradiated, without any decrease in the tumor control rate. In addition to reducing normal tissue toxicity, MRI-LANC-based SBRT provides the opportunity for total dose escalation and subsequent irradiation of a malignant liver tumor.

## 5 Conclusion

As we conclude this editorial, it is becoming evident that, at this point, LINAC-based stereotactic radiotherapy and radiosurgery are not merely treatment opportunities but are becoming a standard of care within the complex landscape of approaches to oncological treatment. Its high conformality and precision, advanced image guidance, and radiobiological effects are transformed into true clinical benefits (in terms of both improved survival and decreased side effects). Many open questions exist, and much ongoing research worldwide underscores their significance. As we look ahead, collaboration, innovation, and patient-centered care will continue, and LINAC-based stereotactic radiotherapy and radiosurgery will take on a robust role in treatment of cancer patients worldwide.

## Author contributions

AK: Writing – original draft. FL: Writing – review & editing.

